# Two Cases of Isolated Ureteral Injury Secondary to Blunt Force Trauma

**DOI:** 10.7759/cureus.10755

**Published:** 2020-10-01

**Authors:** Justin Hughes, Sheree A Bray, Christy Lawson, Bracken Burns

**Affiliations:** 1 Surgery, East Tennessee State University, Quillen College of Medicine, Johnson City, USA

**Keywords:** ureter injury, blunt trauma

## Abstract

Ureteral injuries although rare can cause serious issues. The mechanism of injury is most commonly penetrating but in some rare cases blunt forces can contribute. It is important to diagnose ureteral injuries as soon as possible because they can have significant morbidity and mortality. Here we present two cases of isolated ureteral injury secondary to blunt force trauma. Both patients had the ureteral injury diagnosed by computed tomography (CT) scan and confirmed by a ureterogram with extravasation of contrast. Both patients also had peripelvic cyst, which could have been a contributing risk factor for injury. In both cases, the ureteral injury was repaired using a stent and both patients had no complications. We present these cases along with presentation, diagnostic work-up, and treatment.

## Introduction

Ureteral injury is exceedingly uncommon in the setting of trauma. The incidence of ureteral injury in penetrating trauma is 4% but less than 1% in blunt trauma [1]. A systematic analysis found that ureteral injuries from all types of trauma present with concomitant injuries 90.4% of the time which makes isolated ureteral injuries exceedingly rare [2]. A published incidence of isolated ureteral injuries from blunt trauma could not be found on literature review. Decreased incidence of blunt ureteral injury may be explained by the fact that they are mobile organs and protected by adipose and the psoas muscle. Ureteral injuries, when they do occur in blunt trauma, are seen in rapid deceleration mechanisms such as falls and vehicle collisions. When a ureter injury does occur immediate repair of ureter injury is recommended with complete debridement, tension-free/watertight anastomosis, retroperitoneal drainage, and ureteral stenting [2].

The injury grade can determine the appropriate treatment option for repairing a ureteral injury. The American Association for the Surgery of Trauma (AAST) has developed an injury grading scale for ureteral injuries. Grade I involves hematoma only. Grade II and III are differentiated by whether the laceration involves less than or greater than 50% of the circumference of the ureter. Grade IV and V are characterized by a complete tear of the ureter and distinguished by whether the area of devascularization is less than or greater than 2 cm. A suggested treatment for grade I-III ureteral injuries is placement of a ureteral stent and allowing the ureter to heal over the stent [2]. Location is also an important factor in determining treatment. In one study it was concluded that the upper third of the ureter is more often injured than the middle or lower third. If the injury is to the upper or middle ureter, direct ureteroureterostomy or transureteroureterostomy is recommended. Lower ureter injuries might better be addressed performing ureteral reimplantation (ureteroneocystostomy), psoas hitch, or boari flap [2].

## Case presentation

**Figure 1:**
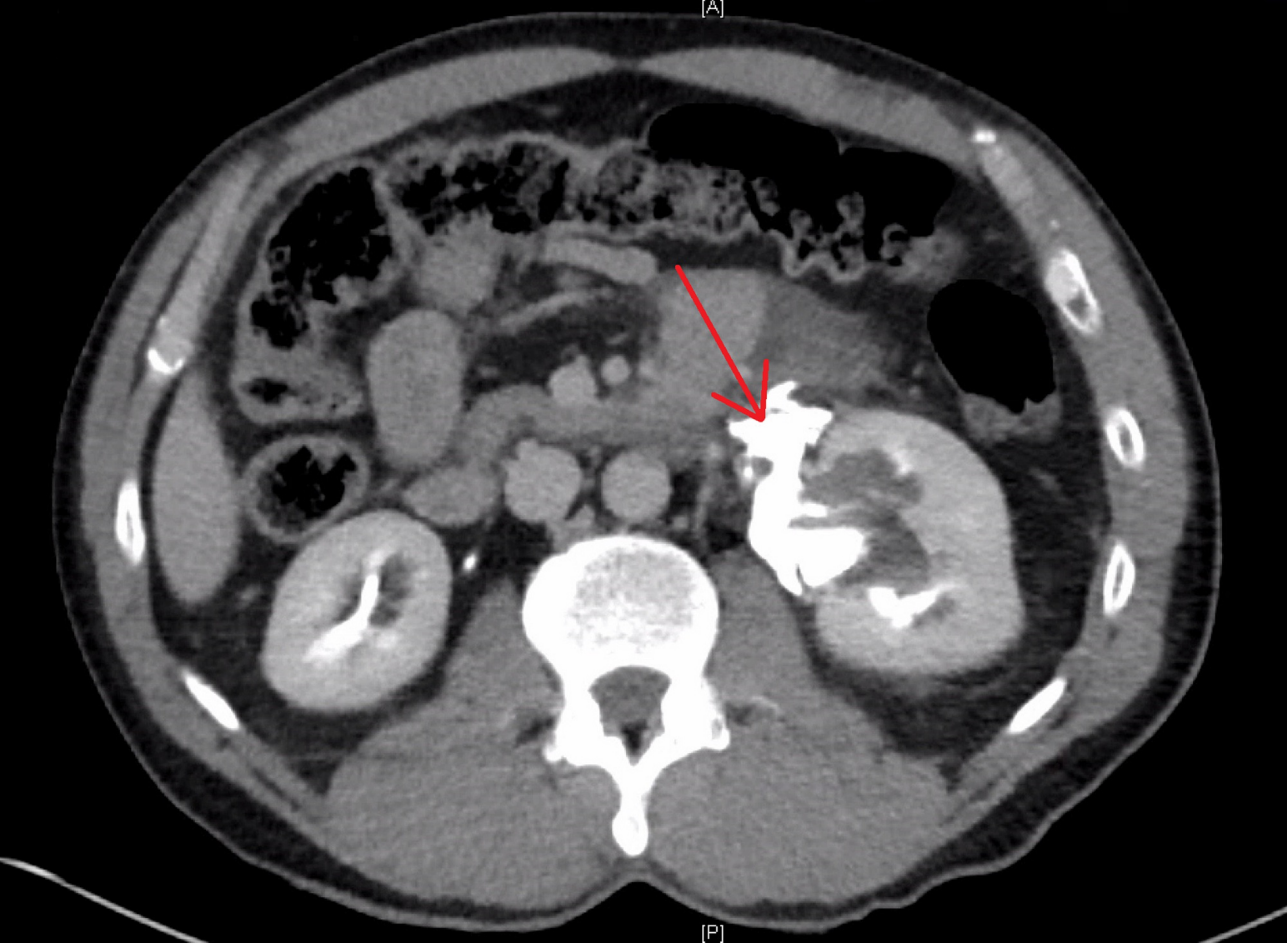
Delayed transverse CT imaging. Area of contrast extravasation denoted by red arrow.

Two cases of blunt ureteral injury without other intraabdominal injuries were seen at our hospital. The first case was a 53-year-old male who presented following a motorcycle collision at approximately 45 miles per hour. He had normal vital signs and cognition. He had a left flank abrasion and tenderness on exam. Frank hematuria was discovered with Foley catheter insertion. Computed tomography (CT) imaging found left clavicle, scapula, and rib fractures with a suspected left ureteral transection with extravasation of contrast around the proximal to mid ureter on delayed imaging (Figure 1) and apparent contrast flow through the distal left ureter into the bladder.

**Figure 2:**
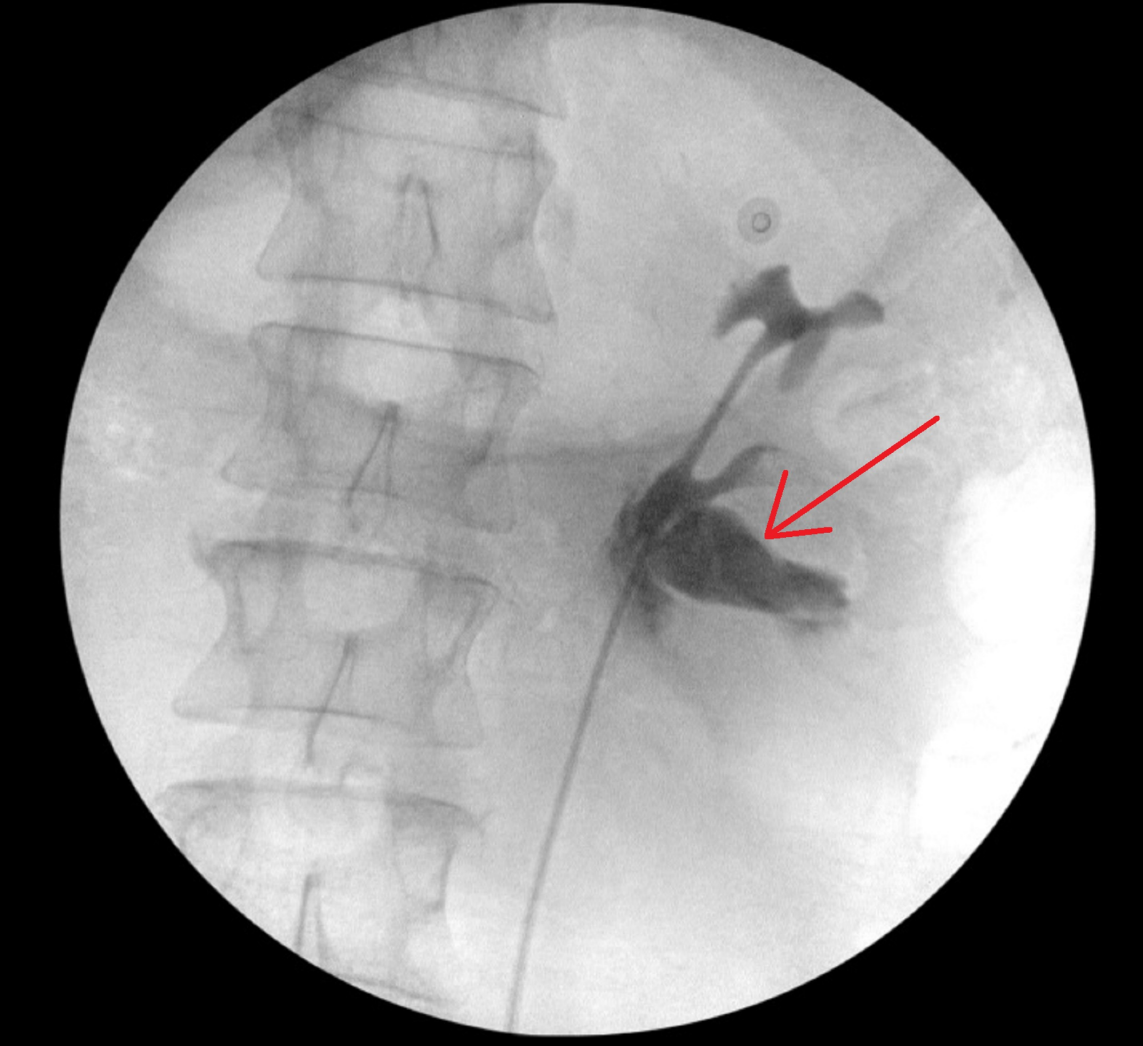
Ureterogram with red arrow denoting contrast extravasation.

Of note he was also found to have peripelvic renal cysts bilaterally. The patient did not have significant medical or surgical history. A ureterogram was performed with extravasation of contrast at the ureteropelvic junction (Figure 2).

A guide wire was able to be passed into the renal pelvis and a stent was placed across the area of ureteral injury in the ureteropelvic junction. The patient had resolution of hematuria after a few days and he had an acute kidney injury preoperatively that resolved prior to discharge.

**Figure 3:**
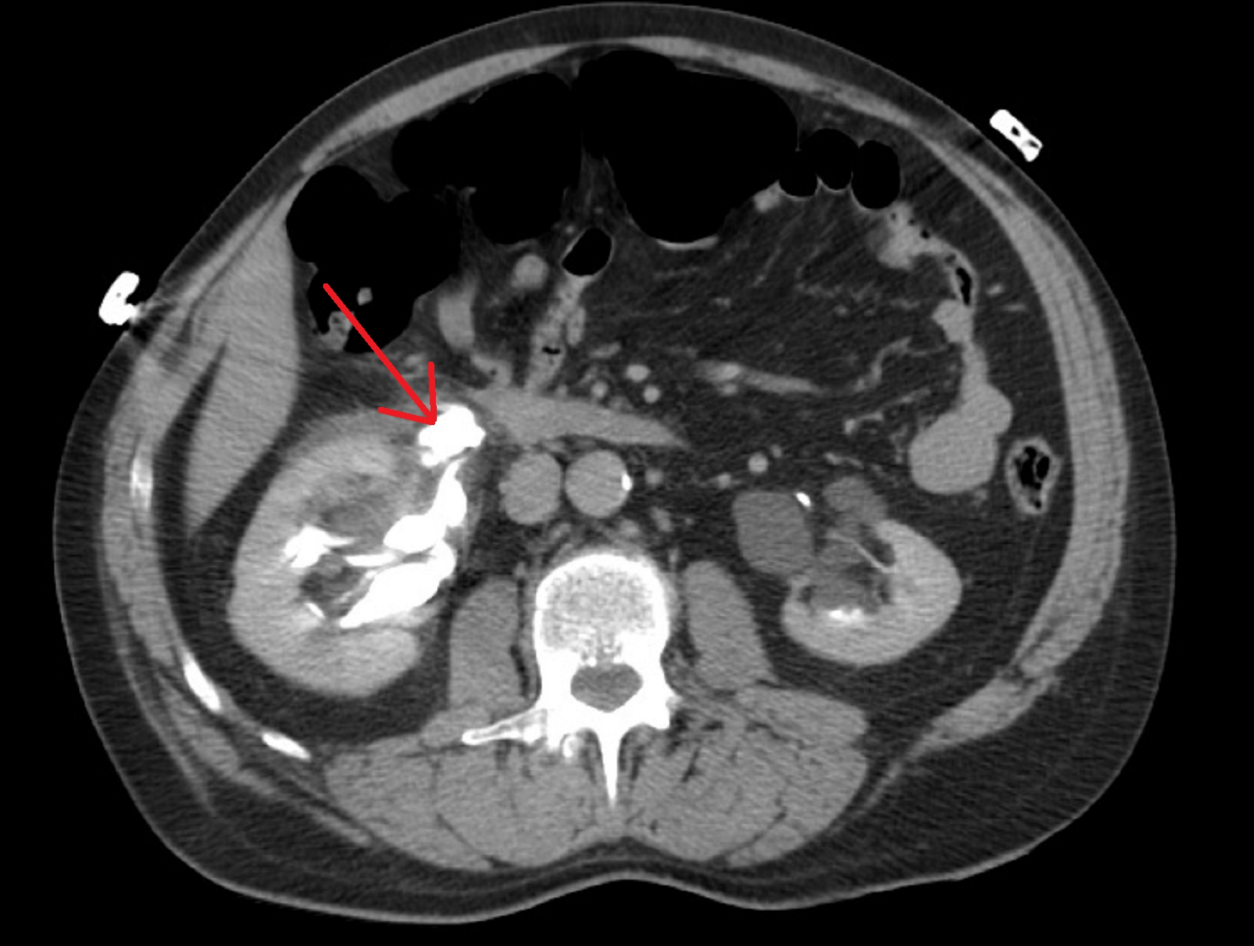
Delayed transverse CT imaging. Area of contrast extravasation denoted by red arrow.

The second case involved a 59-year-old male who slipped and fell while jogging onto his right flank onto concrete. He did not present immediately but was experiencing nausea, vomiting and frank hematuria that made him present approximately 16 hours after his injury to the emergency department. He had normal vital signs and cognition on presentation. Some mild right flank pain was noted but no other injuries were found on further workup. The patient had a history of lithotripsy for a kidney stone, but the side of that procedure was unknown. CT imaging was performed that showed right perirenal contrast extravasation with symmetric uptake of contrast in renal parenchyma (Figure 3).

Of note, bilateral renal peripelvic cysts were seen on imaging. Ureteropelvic junction injury was diagnosed and urgent surgical intervention was recommended to the patient with ureteroscopic stenting or primary repair with stenting. The patient declined further treatment and preferred to follow up with his urologist at an outside facility. He left against medical advice and was lost to follow up.

## Discussion

The two cases discussed both involved isolated ureteral injuries from blunt trauma which is exceedingly rare and after literature search only four case reports were found [3-6]. One of these cases involved a 12-year-old boy following a motor vehicle accident (MVC). The patient sustained a rupture of the right ureter that was immediately diagnosed and repaired. A follow-up intravenous urography was performed eight weeks post surgery and revealed normal ureteral healing with no stricture [3]. With blunt ureteral injuries being rare a high index of suspicion is warranted when evaluating patients. Workup of ureteral injuries is fairly straightforward. Symptoms include nausea, vomiting, flank, and abdominal pain. Peritonitis may be seen if the injury results in urine extravasation into the peritoneal cavity. Approximately 90% of patients with ureteral injury from blunt trauma will have microscopic hematuria [7]. Diagnosis is often made on delayed CT scans with findings of hydronephrosis, contrast extravasation around the ureter and absence of contrast in the distal ureter in the case of complete disruption. Ultrasound can also be used to evaluate for ureteral injury as it can visualize hydronephrosis and free fluid around the ureter. Radionuclide scans can evaluate urine flow through the ureters and are often used following surgical repair [7]. Ureter injuries can be graded according to the American Association for Surgery of Trauma with Grade I being a contusion or hematoma without devascularization, Grade II laceration with less than 50% transection, Grade III laceration with greater than 50% transection, Grade IV complete transection with less than 2 cm of devascularization, and Grade V being avulsion with >2 cm devascularization [8]. Injury grades are rarely reported because it requires direct visualization. Once diagnosis of a ureteral injury is made surgical correction is recommended. Small partial injuries may be able to heal with ureteroscopic stent placement alone otherwise larger injuries are approached through different methods based on location. A primary repair with debridement of devitalized tissue, a tension-free anastomosis with spatulation of both ends and retroperitoneal drainage can be performed in any location if enough ureter length is available [9]. Injuries in the distal third of the ureter may be repaired with implantation into the bladder or transureteroureterostomy. Middle third injuries may require a transureteroureterostomy and a downward nephropexy can be used to relieve any anastomotic tension. Injuries to the upper third may require bowel replacement of the ureter or kidney autotransplantation if a primary anastomosis is not feasible [7]. If a ureteral injury is left undiagnosed complications may occur including urinoma formation, periureteral abscess, ureteral fistula, or ureteral stricture [10]. Delayed diagnosis often results in a hostile operative field and diversion of urine with a nephrostomy tube and/or stent placement with delayed surgical repair is often necessary [9].

The two cases presented although not formally graded were most likely grade II or grade III injuries. The first case was treated successfully with ureteroscopic stent placement. The plan for the second patient was also for ureteroscopic stent placement versus primary surgical repair with stent placement but the patient elected to leave AMA.

## Conclusions

Ureteral injuries from blunt trauma remain rare and not much is known of the etiology. It is possible that patients may have risk factors that predispose them to a ureteral injury. In the two cases discussed above both patients had peripelvic renal cysts which may have created a fulcrum for torque to be applied at the ureteropelvic junction and result in injury. It is also possible that the same cause of the cysts may cause weakness of ureter tissues and predispose to ureteral injury. Overall, much is still left to discover about the rare phenomenon of blunt ureteral injuries.
